# 3,5-Dinitro­pyridin-4(1*H*)-one monohydrate

**DOI:** 10.1107/S1600536808024604

**Published:** 2008-08-06

**Authors:** Ying Li, Ping Li, Qiu-Ping Zhou, Guo-Fang Zhang, Seik Weng Ng

**Affiliations:** a Chemistry Department, Jingning Normal College, Wulanchabu, Inner Mongolia 012000, People’s Republic of China; b School of Chemistry and Materials Science, Shaanxi Normal University, Xi’an 710062, People’s Republic of China; c Department of Chemistry, University of Malaya, 50603 Kuala Lumpur, Malaysia

## Abstract

The three independent organic mol­ecules of 3,5-dinitro­pyridin-4(1*H*)-one monohydrate, C_5_H_3_N_3_O_5_·H_2_O, each feature an N—H⋯O_water_ hydrogen bond. Each water mol­ecule serves as hydrogen-bond donor to two carbonyl O atoms; these hydrogen bonds give rise to a layer motif. Two of the three formula units lie on special positions of site symmetry 2.

## Related literature

The parent pyridin-4-one homolog crystallizes with five pyridone and six water mol­ecules in the asymmetric unit; see: Jones (2001 ▶).
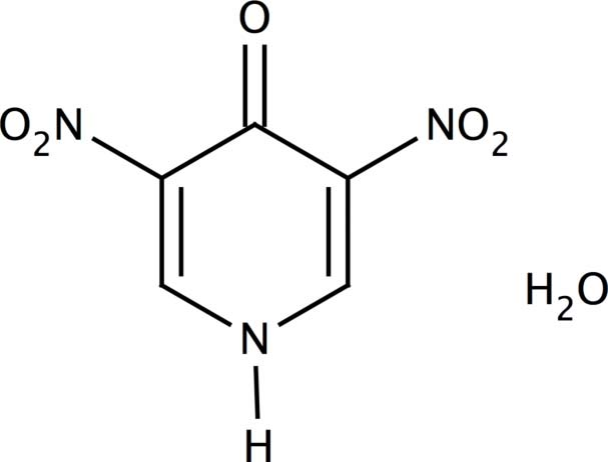


## Experimental

### 

#### Crystal data

C_5_H_3_N_3_O_5_·H_2_O
*M* *_r_* = 203.12
Orthorhombic, 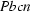

*a* = 21.728 (2) Å
*b* = 21.654 (2) Å
*c* = 6.5713 (5) Å
*V* = 3091.7 (4) Å^3^
*Z* = 16
Mo *K*α radiation
μ = 0.16 mm^−1^
*T* = 293 (2) K
0.45 × 0.45 × 0.20 mm

#### Data collection

Bruker APEXII diffractometer
Absorption correction: none
21800 measured reflections
3555 independent reflections
2852 reflections with *I* > 2σ(*I*)
*R*_int_ = 0.022

#### Refinement

*R*[*F* ^2^ > 2σ(*F* ^2^)] = 0.040
*wR*(*F* ^2^) = 0.123
*S* = 1.08
3555 reflections
281 parameters
10 restraints
H atoms treated by a mixture of independent and constrained refinement
Δρ_max_ = 0.34 e Å^−3^
Δρ_min_ = −0.31 e Å^−3^

### 

Data collection: *APEX2* (Bruker, 2007 ▶); cell refinement: *SAINT* (Bruker, 2007 ▶); data reduction: *SAINT*; program(s) used to solve structure: *SHELXS97* (Sheldrick, 2008 ▶); program(s) used to refine structure: *SHELXL97* (Sheldrick, 2008 ▶); molecular graphics: *X-SEED* (Barbour, 2001 ▶); software used to prepare material for publication: *publCIF* (Westrip, 2008 ▶).

## Supplementary Material

Crystal structure: contains datablocks global, I. DOI: 10.1107/S1600536808024604/pk2110sup1.cif

Structure factors: contains datablocks I. DOI: 10.1107/S1600536808024604/pk2110Isup2.hkl

Additional supplementary materials:  crystallographic information; 3D view; checkCIF report

## Figures and Tables

**Table 1:** Hydrogen-bond geometry (Å, °)

*D*—H⋯*A*	*D*—H	H⋯*A*	*D*⋯*A*	*D*—H⋯*A*
N3—H3⋯O1w	0.85 (1)	1.86 (1)	2.703 (2)	172 (2)
N5—H5⋯O2w	0.85 (1)	1.84 (1)	2.692 (3)	180
N6—H6⋯O3w	0.85 (1)	1.87 (1)	2.723 (3)	180
O1w—H11⋯O8^i^	0.85 (1)	2.04 (1)	2.878 (2)	168 (2)
O1w—H12⋯O11^ii^	0.86 (1)	2.02 (1)	2.866 (2)	168 (2)
O2w—H21⋯O3^iii^	0.84 (1)	2.05 (1)	2.888 (2)	173 (1)
O3w—H31⋯O3	0.84 (1)	2.05 (1)	2.890 (2)	172 (2)

Symmetry codes: (i) 
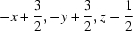
; (ii) 
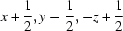
; (iii) 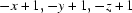.

**Fig. 1.**
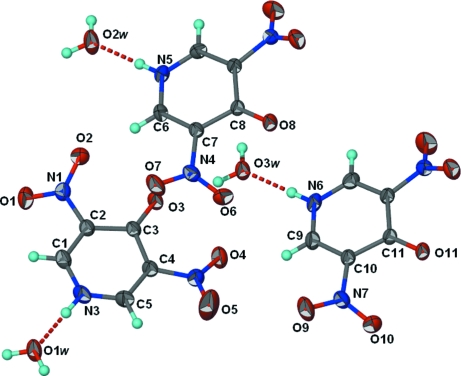
Thermal ellipsoid plot (Barbour, 2001) plot of the three independent molecules of C5H3N3O5.H2O at the 50% probability level. Hydrogen atoms are drawn as spheres of arbitrary radius.

## supplementary crystallographic information

### Comment

3,5-Dinitro-4-pyridinol, a specialty chemical, is assumed in chemical catalogs to exist in the enol form. The homolog, 4-pyridinol, is in fact 4-pyridinone^.^6/5hydrate.  It has five independent pyridone and six water molecules that are hydrogen bonded to form layers (Jones, 2001). The presence of two electron-withdrawing groups in the title compound should enhance its propensity to form hydrogen bonds, and this is borne out in the present study. The three independent molecules of 3,5-dinitro-1*H*-pyridin-4-one hydrate (Fig. 1) each feature an N–H···O_water_ hydrogen bond; each water molecule serves as hydrogen-bond donor to two carbonyl oxygen atoms, and these hydrogen bonds give rise to a layered structure.

### Experimental

4-Hydroxy-3-pyridine (19 g, 0.02 mol) was dissolved in fuming sulfuric acid (50% by SO_3_ content) (60 ml), and to the solution was added an oleum-fuming nitric acid (1/3) mixture (50 ml). The temperature was kept at 0°C for an hour. The temperature was raised to 413 K over a period of one hour, and then held at 403 K for another 16 h. The mixture was poured into ice (200 g) to quench the reaction. Some 27 g of material was isolated. Crystals were obtained by recrystallization from water.

### Refinement

Carbon-bound H-atoms were placed in calculated positions (C—H 0.93 Å) and were included in the refinement using a riding model approximation, with *U*(H) 1.2*U*_eq_(C). The amino and water H-atoms were refined with distance restraints of O–H = N–H 0.85 (1) and H···H 1.39 (1) Å; their temperature factors *U*_iso_ were freely refined.

### Crystal data

**Table:**

C_5_H_3_N_3_O_5_·H_2_O	*F*_000_ = 1664
*M**_r_* = 203.12	*D*_x_ = 1.746 Mg m^−^^3^
Orthorhombic, *P**b**c**n*	Mo *K*α radiation λ = 0.71073 Å
Hall symbol: -P 2n 2ab	Cell parameters from 6494 reflections
*a* = 21.728 (2) Å	θ = 2.7–27.9º
*b* = 21.654 (2) Å	µ = 0.16 mm^−^^1^
*c* = 6.5713 (5) Å	*T* = 293 (2) K
*V* = 3091.7 (4) Å^3^	Block, colorless
*Z* = 16	0.45 × 0.45 × 0.20 mm

### Data collection

**Table:**

Bruker APEXII diffractometer	3555 independent reflections
Radiation source: fine-focus sealed tube	2852 reflections with *I* > 2σ(*I*)
Monochromator: graphite	*R*_int_ = 0.022
Detector resolution: 9 pixels mm^-1^	θ_max_ = 27.5º
*T* = 293(2) K	θ_min_ = 2.7º
φ and ω scans	*h* = −28→27
Absorption correction: none	*k* = −28→27
21800 measured reflections	*l* = −8→8

### Refinement

**Table:**

Refinement on *F*^2^	Secondary atom site location: difference Fourier map
Least-squares matrix: full	Hydrogen site location: inferred from neighbouring sites
*R*[*F*^2^ > 2σ(*F*^2^)] = 0.040	H atoms treated by a mixture of independent and constrained refinement
*wR*(*F*^2^) = 0.123	*w* = 1/[σ^2^(*F*_o_^2^) + (0.0528*P*)^2^ + 1.6904*P*] where *P* = (*F*_o_^2^ + 2*F*_c_^2^)/3
*S* = 1.08	(Δ/σ)_max_ < 0.001
3555 reflections	Δρ_max_ = 0.35 e Å^−^^3^
281 parameters	Δρ_min_ = −0.31 e Å^−^^3^
10 restraints	Extinction correction: none
Primary atom site location: structure-invariant direct methods	

### Fractional atomic coordinates and isotropic or equivalent isotropic displacement parameters (Å^2^)

**Table:**

	*x*	*y*	*z*	*U*_iso_*/*U*_eq_	
O1	0.69961 (7)	0.46193 (6)	0.2892 (3)	0.0552 (4)	
O2	0.62136 (6)	0.50982 (6)	0.4163 (3)	0.0464 (4)	
O3	0.60319 (6)	0.62125 (6)	0.2292 (2)	0.0431 (4)	
O4	0.61633 (6)	0.73545 (6)	0.0695 (3)	0.0486 (4)	
O5	0.70310 (8)	0.77792 (8)	0.1081 (6)	0.1176 (12)	
O6	0.60858 (6)	0.75858 (6)	0.5467 (3)	0.0496 (4)	
O7	0.65774 (6)	0.67898 (7)	0.6579 (3)	0.0562 (4)	
O8	0.5000	0.77618 (7)	0.7500	0.0389 (4)	
O9	0.65904 (7)	0.92189 (7)	0.1991 (3)	0.0659 (5)	
O10	0.61401 (6)	1.00203 (6)	0.0759 (3)	0.0482 (4)	
O11	0.5000	1.02039 (7)	0.2500	0.0357 (4)	
O1W	0.91543 (6)	0.62174 (6)	0.2293 (3)	0.0507 (4)	
O2W	0.5000	0.46255 (9)	0.7500	0.0627 (7)	
O3W	0.5000	0.70522 (8)	0.2500	0.0416 (5)	
N1	0.67114 (7)	0.50953 (7)	0.3278 (3)	0.0360 (3)	
N2	0.67066 (7)	0.73271 (7)	0.1089 (3)	0.0435 (4)	
N3	0.79137 (7)	0.61956 (8)	0.1992 (3)	0.0434 (4)	
N4	0.61008 (6)	0.70807 (7)	0.6311 (3)	0.0357 (3)	
N5	0.5000	0.58686 (9)	0.7500	0.0376 (5)	
N6	0.5000	0.83100 (9)	0.2500	0.0384 (5)	
N7	0.61264 (7)	0.95139 (7)	0.1582 (3)	0.0377 (4)	
C1	0.76100 (8)	0.56854 (9)	0.2549 (3)	0.0390 (4)	
H1A	0.7829	0.5329	0.2857	0.047*	
C2	0.69872 (8)	0.56805 (8)	0.2672 (3)	0.0315 (4)	
C3	0.65995 (8)	0.62114 (7)	0.2208 (3)	0.0302 (4)	
C4	0.69804 (8)	0.67347 (8)	0.1610 (3)	0.0337 (4)	
C5	0.76066 (8)	0.67135 (9)	0.1539 (3)	0.0405 (4)	
H5A	0.7825	0.7065	0.1169	0.049*	
C6	0.55166 (8)	0.61756 (8)	0.7027 (3)	0.0345 (4)	
H6A	0.5874	0.5957	0.6733	0.041*	
C7	0.55244 (7)	0.68038 (7)	0.6972 (3)	0.0290 (4)	
C8	0.5000	0.71922 (10)	0.7500	0.0269 (5)	
C9	0.55223 (9)	0.86172 (8)	0.2128 (3)	0.0360 (4)	
H9	0.5884	0.8398	0.1893	0.043*	
C10	0.55324 (8)	0.92451 (7)	0.2090 (3)	0.0301 (4)	
C11	0.5000	0.96327 (10)	0.2500	0.0278 (5)	
H11	0.9376 (8)	0.6540 (6)	0.220 (4)	0.059 (7)*	
H12	0.9385 (8)	0.5898 (6)	0.218 (4)	0.053 (7)*	
H21	0.4680 (2)	0.4409 (7)	0.757 (4)	0.055 (7)*	
H31	0.5318 (3)	0.6832 (7)	0.236 (4)	0.058 (7)*	
H3	0.8306 (5)	0.6181 (10)	0.199 (4)	0.057 (7)*	
H5	0.5000	0.5477 (5)	0.7500	0.051 (9)*	
H6	0.5000	0.7915 (5)	0.2500	0.051 (9)*	

### Atomic displacement parameters (Å^2^)

**Table:**

	*U*^11^	*U*^22^	*U*^33^	*U*^12^	*U*^13^	*U*^23^
O1	0.0543 (9)	0.0322 (7)	0.0790 (12)	0.0112 (6)	0.0045 (8)	0.0018 (7)
O2	0.0369 (7)	0.0417 (7)	0.0607 (9)	−0.0020 (6)	0.0068 (7)	0.0077 (7)
O3	0.0218 (6)	0.0344 (7)	0.0730 (10)	0.0014 (5)	0.0029 (6)	0.0061 (6)
O4	0.0381 (7)	0.0401 (7)	0.0676 (10)	0.0086 (6)	−0.0039 (7)	0.0016 (7)
O5	0.0504 (10)	0.0435 (10)	0.259 (4)	−0.0121 (8)	−0.0082 (15)	0.0441 (15)
O6	0.0415 (7)	0.0392 (7)	0.0681 (10)	−0.0090 (6)	0.0128 (7)	0.0059 (7)
O7	0.0273 (7)	0.0575 (9)	0.0837 (12)	0.0076 (6)	0.0053 (7)	−0.0044 (8)
O8	0.0326 (9)	0.0211 (8)	0.0631 (13)	0.000	0.0069 (8)	0.000
O9	0.0304 (7)	0.0501 (9)	0.1171 (16)	0.0104 (6)	0.0009 (8)	0.0031 (9)
O10	0.0399 (7)	0.0372 (7)	0.0673 (10)	−0.0056 (6)	0.0073 (7)	0.0088 (7)
O11	0.0305 (9)	0.0199 (8)	0.0567 (12)	0.000	0.0020 (8)	0.000
O1W	0.0263 (7)	0.0294 (7)	0.0962 (13)	0.0008 (5)	−0.0054 (7)	0.0013 (7)
O2W	0.0296 (10)	0.0253 (9)	0.133 (2)	0.000	−0.0014 (12)	0.000
O3W	0.0286 (9)	0.0244 (8)	0.0719 (14)	0.000	0.0004 (9)	0.000
N1	0.0345 (8)	0.0322 (8)	0.0414 (9)	0.0028 (6)	−0.0042 (7)	0.0014 (6)
N2	0.0356 (8)	0.0335 (8)	0.0614 (11)	−0.0008 (6)	0.0060 (8)	0.0067 (7)
N3	0.0206 (7)	0.0488 (10)	0.0608 (11)	0.0008 (6)	−0.0006 (7)	0.0012 (8)
N4	0.0275 (7)	0.0353 (8)	0.0442 (9)	−0.0025 (6)	0.0039 (6)	−0.0084 (7)
N5	0.0434 (12)	0.0192 (9)	0.0503 (14)	0.000	0.0058 (10)	0.000
N6	0.0460 (13)	0.0188 (9)	0.0504 (13)	0.000	0.0006 (10)	0.000
N7	0.0293 (7)	0.0333 (8)	0.0505 (10)	0.0011 (6)	0.0040 (7)	−0.0052 (7)
C1	0.0282 (9)	0.0412 (10)	0.0476 (11)	0.0067 (7)	−0.0031 (8)	−0.0008 (8)
C2	0.0270 (8)	0.0305 (8)	0.0371 (9)	0.0010 (6)	−0.0011 (7)	−0.0017 (7)
C3	0.0237 (8)	0.0296 (8)	0.0374 (9)	0.0006 (6)	0.0012 (7)	−0.0023 (7)
C4	0.0264 (8)	0.0324 (9)	0.0424 (10)	0.0001 (7)	0.0008 (7)	0.0007 (7)
C5	0.0288 (9)	0.0410 (10)	0.0517 (12)	−0.0062 (7)	0.0027 (8)	0.0011 (9)
C6	0.0339 (9)	0.0289 (8)	0.0406 (10)	0.0063 (7)	0.0034 (7)	−0.0019 (7)
C7	0.0261 (8)	0.0269 (8)	0.0339 (9)	−0.0002 (6)	0.0009 (7)	−0.0015 (6)
C8	0.0258 (11)	0.0233 (10)	0.0316 (12)	0.000	−0.0020 (9)	0.000
C9	0.0387 (10)	0.0270 (8)	0.0421 (10)	0.0055 (7)	0.0003 (8)	−0.0018 (7)
C10	0.0296 (8)	0.0239 (8)	0.0369 (9)	0.0001 (6)	−0.0006 (7)	−0.0009 (6)
C11	0.0281 (11)	0.0228 (10)	0.0323 (12)	0.000	−0.0021 (9)	0.000

### Geometric parameters (Å, °)

**Table:**

O1—N1	1.2285 (19)	N5—C6	1.341 (2)
O2—N1	1.228 (2)	N5—C6^i^	1.341 (2)
O3—C3	1.235 (2)	N5—H5	0.848 (10)
O4—N2	1.210 (2)	N6—C9	1.338 (2)
O5—N2	1.206 (2)	N6—C9^ii^	1.338 (2)
O6—N4	1.227 (2)	N6—H6	0.854 (10)
O7—N4	1.225 (2)	N7—C10	1.455 (2)
O8—C8	1.233 (3)	C1—C2	1.356 (2)
O9—N7	1.223 (2)	C1—H1A	0.9300
O10—N7	1.223 (2)	C2—C3	1.457 (2)
O11—C11	1.237 (3)	C3—C4	1.457 (2)
O1W—H11	0.851 (9)	C4—C5	1.362 (2)
O1W—H12	0.858 (9)	C5—H5A	0.9300
O2W—H21	0.840 (8)	C6—C7	1.361 (2)
O3W—H31	0.844 (8)	C6—H6A	0.9300
N1—C2	1.457 (2)	C7—C8	1.458 (2)
N2—C4	1.455 (2)	C8—C7^i^	1.458 (2)
N3—C1	1.338 (3)	C9—C10	1.360 (2)
N3—C5	1.338 (3)	C9—H9	0.9300
N3—H3	0.853 (10)	C10—C11	1.454 (2)
N4—C7	1.455 (2)	C11—C10^ii^	1.454 (2)
			
H11—O1W—H12	109.1 (14)	O3—C3—C4	125.31 (15)
O2—N1—O1	123.08 (16)	O3—C3—C2	124.72 (15)
O2—N1—C2	119.15 (14)	C4—C3—C2	109.97 (14)
O1—N1—C2	117.76 (15)	C5—C4—N2	115.48 (16)
O5—N2—O4	121.96 (17)	C5—C4—C3	123.38 (16)
O5—N2—C4	118.53 (17)	N2—C4—C3	121.12 (15)
O4—N2—C4	119.51 (15)	N3—C5—C4	121.24 (17)
C1—N3—C5	120.47 (16)	N3—C5—H5A	119.4
C1—N3—H3	117.6 (16)	C4—C5—H5A	119.4
C5—N3—H3	121.8 (16)	N5—C6—C7	120.80 (16)
O7—N4—O6	123.11 (15)	N5—C6—H6A	119.6
O7—N4—C7	118.20 (15)	C7—C6—H6A	119.6
O6—N4—C7	118.66 (14)	C6—C7—N4	115.51 (15)
C6—N5—C6^i^	120.6 (2)	C6—C7—C8	124.09 (16)
C6—N5—H5	119.71 (10)	N4—C7—C8	120.39 (14)
C6^i^—N5—H5	119.71 (10)	O8—C8—C7^i^	125.23 (9)
C9—N6—C9^ii^	120.4 (2)	O8—C8—C7	125.23 (9)
C9—N6—H6	119.82 (11)	C7^i^—C8—C7	109.54 (19)
C9^ii^—N6—H6	119.82 (10)	N6—C9—C10	120.94 (17)
O10—N7—O9	123.05 (16)	N6—C9—H9	119.5
O10—N7—C10	118.79 (14)	C10—C9—H9	119.5
O9—N7—C10	118.16 (16)	C9—C10—C11	124.11 (16)
N3—C1—C2	120.99 (17)	C9—C10—N7	114.74 (15)
N3—C1—H1A	119.5	C11—C10—N7	121.15 (14)
C2—C1—H1A	119.5	O11—C11—C10^ii^	125.25 (9)
C1—C2—N1	115.69 (15)	O11—C11—C10	125.25 (9)
C1—C2—C3	123.94 (16)	C10^ii^—C11—C10	109.50 (19)
N1—C2—C3	120.36 (14)		
			
C5—N3—C1—C2	−0.1 (3)	C6^i^—N5—C6—C7	−1.47 (13)
N3—C1—C2—N1	179.48 (18)	N5—C6—C7—N4	−176.15 (14)
N3—C1—C2—C3	0.5 (3)	N5—C6—C7—C8	3.0 (3)
O2—N1—C2—C1	152.19 (18)	O7—N4—C7—C6	−26.7 (2)
O1—N1—C2—C1	−27.0 (3)	O6—N4—C7—C6	151.55 (18)
O2—N1—C2—C3	−28.8 (3)	O7—N4—C7—C8	154.12 (15)
O1—N1—C2—C3	151.95 (18)	O6—N4—C7—C8	−27.7 (2)
C1—C2—C3—O3	179.23 (19)	C6—C7—C8—O8	178.50 (13)
N1—C2—C3—O3	0.3 (3)	N4—C7—C8—O8	−2.34 (18)
C1—C2—C3—C4	−0.1 (3)	C6—C7—C8—C7^i^	−1.50 (13)
N1—C2—C3—C4	−179.00 (16)	N4—C7—C8—C7^i^	177.66 (18)
O5—N2—C4—C5	−16.1 (3)	C9^ii^—N6—C9—C10	−0.98 (13)
O4—N2—C4—C5	164.46 (19)	N6—C9—C10—C11	2.0 (3)
O5—N2—C4—C3	162.5 (3)	N6—C9—C10—N7	−177.52 (15)
O4—N2—C4—C3	−16.9 (3)	O10—N7—C10—C9	152.08 (18)
O3—C3—C4—C5	179.9 (2)	O9—N7—C10—C9	−27.5 (3)
C2—C3—C4—C5	−0.7 (3)	O10—N7—C10—C11	−27.5 (2)
O3—C3—C4—N2	1.4 (3)	O9—N7—C10—C11	152.96 (17)
C2—C3—C4—N2	−179.23 (17)	C9—C10—C11—O11	179.01 (13)
C1—N3—C5—C4	−0.7 (3)	N7—C10—C11—O11	−1.48 (19)
N2—C4—C5—N3	179.75 (19)	C9—C10—C11—C10^ii^	−0.99 (13)
C3—C4—C5—N3	1.2 (3)	N7—C10—C11—C10^ii^	178.52 (19)

Symmetry codes: (i) −*x*+1, *y*, −*z*+3/2; (ii) −*x*+1, *y*, −*z*+1/2.

### Hydrogen-bond geometry (Å, °)

**Table:**

*D*—H···*A*	*D*—H	H···*A*	*D*···*A*	*D*—H···*A*
N3—H3···O1w	0.85 (1)	1.86 (1)	2.703 (2)	172 (2)
N5—H5···O2w	0.85 (1)	1.84 (1)	2.692 (3)	180
N6—H6···O3w	0.85 (1)	1.87 (1)	2.723 (3)	180
O1w—H11···O8^iii^	0.85 (1)	2.04 (1)	2.878 (2)	168 (2)
O1w—H12···O11^iv^	0.86 (1)	2.02 (1)	2.866 (2)	168 (2)
O2w—H21···O3^v^	0.84 (1)	2.05 (1)	2.888 (2)	173 (1)
O3w—H31···O3	0.84 (1)	2.05 (1)	2.890 (2)	172 (2)

Symmetry codes: (iii) −*x*+3/2, −*y*+3/2, *z*−1/2; (iv) *x*+1/2, *y*−1/2, −*z*+1/2; (v) −*x*+1, −*y*+1, −*z*+1.
